# Heat Stress and Bull Semen: From Endocrine and Molecular Dysregulation to Targeted Intervention

**DOI:** 10.3390/biom16101481

**Published:** 2026-10-12

**Authors:** Qingyu Zhu, Wei You, Haijian Cheng, Xin Hu, Fugui Jiang, Luyang Sun, Enliang Song

**Affiliations:** 1 Shandong Provincial Key Laboratory of Livestock and Poultry Breeding, Institute of Poultry Science, Shandong Academy of Agricultural Sciences, Jinan 250100, China; zhuqingyu@saas.ac.cn (Q.Z.); sunluyang@saas.ac.cn (L.S.); 2 Key Laboratory of Livestock and Poultry Multi-Omics of MARA, Institute of Animal Science and Veterinary Medicine, Shandong Academy of Agricultural Sciences, Jinan 250100, China; youwei@saas.ac.cn (W.Y.); chenghaijian@saas.ac.cn (H.C.); huxin@saas.ac.cn (X.H.); jiangfugui@saas.ac.cn (F.J.)

**Keywords:** heat stress, bull, semen quality, neuroendocrine, epigenetic, “gut–testis” axis, mitigation strategy

## Abstract

Heat stress, a critical environmental stressor intensified by global warming, increasingly threatens bull semen quality. This review synthesizes the multi-level mechanisms underlying this impairment. Heat-stressed bulls exhibit behavioral changes that serve as early indicators of reproductive dysfunction, alongside pronounced declines in routine semen parameters and alterations in the seminal plasma biochemical microenvironment. At the endocrine level, heat stress disrupts the hypothalamic–pituitary axes, suppressing testosterone synthesis and perturbing the testicular microenvironment to compromise spermatogenesis. At the molecular level, altered expression of heat-stress-related genes and epigenetic reprogramming—involving non-coding RNAs, DNA methylation, and m^6^A methylation—collectively disturb this process. Notably, the “gut–testis” axis emerges as a key pathway: heat-induced gut microbiota dysbiosis compromises intestinal barrier integrity, and immune and endocrine signals from translocated microbial metabolites ultimately impair testicular function and semen quality, as evidenced by cross-species studies. At the application level, the above mechanistic evidence, which is largely derived from model animals or non-bovine livestock, still requires direct and systematic validation in bulls. Collectively, this review provides an integrative mechanistic framework for heat stress-induced deterioration of bull semen quality and highlights critical directions for developing targeted mitigation strategies under global climate change.

## 1. Introduction

With the intensification of global warming and the increasing frequency of extreme high-temperature events, summer heat poses unprecedented challenges to the high-quality development of the livestock industry. Heat stress has emerged as a primary limiting factor constraining the breeding and reproductive productivity of livestock and poultry, exerting adverse effects on production stability and germplasm improvement. In the cattle industry, heat stress not only induces a series of behavioral abnormalities, such as reduced feed intake and altered activity, but also severely disrupts the body’s physiological metabolism and reproductive function [1,2]. Among these, the impairment of reproductive function in breeding bulls poses a particularly notable threat to the germplasm improvement stage, and the resulting direct and indirect economic losses should not be underestimated.

As a cornerstone of modern cattle breeding and genetic improvement programs, breeding bulls serve as the primary vectors of genetic material and breeding value. The quality of their semen is a critical determinant of herd reproductive efficiency and the pace of genetic gain [3]. Leveraging artificial insemination (AI) technology, a single elite sire can inseminate tens of thousands of cows annually, playing an indispensable role in enhancing productivity and driving genetic progress across both beef and dairy sectors [4,5]. Consequently, maintaining stable semen quality and mitigating heat stress-mediated reproductive impairments in breeding bulls are paramount for ensuring sustainable, high-quality output in the cattle breeding industry and accelerating the selection of superior genetic lines.

The impact of heat stress on bull semen quality is typically manifested as a predictable decline in key parameters, such as reduced sperm motility and concentration, along with an elevated rate of morphological abnormalities. This effect exhibits distinct seasonal characteristics, with the most severe impairment occurring during the high-temperature and high-humidity periods of summer, followed by a gradual, natural recovery during the winter and spring seasons [6,7]. Drawing on the experience described above, large-scale bull studs typically have to appropriately lower their semen collection intensity under high summer temperatures. This reduction not only diminishes the annual output of high-quality frozen semen but also, to a certain degree, hampers the dissemination of elite genetic resources and the advancement of the breeding program. Currently, research on the effects of heat stress on bull reproductive performance has evolved from macroscopic phenotypic observations to mechanistic exploration at the molecular level. Early studies primarily focused on behavioral alterations and seasonal fluctuations in semen parameters. Contemporary research, however, predominantly investigates systemic oxidative stress damage, neuroendocrine dysregulation, and—most notably—the inhibition of testosterone synthesis and testicular microenvironment impairment mediated by the disruption of the classic “hypothalamic–pituitary–gonadal (HPG) axis.” Furthermore, researchers are progressively uncovering molecular regulatory patterns, including gene expression regulation and epigenetic reprogramming [8,9]. Additionally, the association between gut microbiota and the reproductive system is increasingly being substantiated, and the regulatory role of the “gut–testis” axis provides a novel perspective for elucidating the mechanisms underlying heat stress-induced reproductive impairment [10].

Building on previous reviews of heat stress and bovine reproduction, this review provides an integrated, multi-level framework spanning behavioral responses, conventional semen parameters, the seminal microenvironment, oxidative stress injury, neu-roendocrine disruption, and epigenomic remodeling, while also incorporating the emerging “gut–testis” axis as a novel regulatory dimension. Furthermore, this review critically evaluates the strength and consistency of available evidence—explicitly distinguishing between direct findings in bulls and mechanistic support from other species—and highlights areas where the evidence remains preliminary or inconsistent. Potential intervention strategies, including nutritional modulation and genetic breeding, are also discussed. By synthesizing these diverse lines of evidence, this work aims to refine the theoretical framework underlying heat stress-induced reproductive damage in bulls, identify key knowledge gaps, and provide a scientific basis for developing targeted mitigation strategies under climate change.

## 2. Literature Search Strategy

The present review integrates evidence on the effects of heat stress on semen traits in bulls and the underlying molecular mechanisms. Literature searches were performed in the PubMed database using the following keywords and their combinations: heat stress; bull; semen quality; neuroendocrine; miRNA; circRNA; lncRNA; DNA methylation, m^6^A methylation, gut microbiota; “gut–testis” axis; gene editing. The search covered English-language articles published up to 20 September 2026. Articles were prioritized based on methodological rigor, relevance to the review’s scope, and quality of evidence rather than solely on journal indexing status. Literature not published in English was not included in the primary search; however, the authors acknowledge that this restriction may have resulted in the omission of relevant studies, particularly those published in other languages in the field of veterinary and animal science. Studies matching the scope of the present review were read in full, after which their inclusion was determined by further assessment of methodological relevance and information value.

The studies discussed in this review employ different heat stress models, primarily including natural environmental heat exposure (e.g., seasonal heat stress in bulls) and in vitro hyperthermia of cultured cells. These models differ in their ecological validity and the level of inference they support: environmental exposure captures the integrated organismal response but lacks experimental control, whereas in vitro hyperthermia enables mechanistic dissection but removes systemic interactions. The experimental model is identified where applicable throughout the text.

Given that direct evidence on the molecular mechanisms underlying heat stress-induced spermatogenic dysfunction in bulls remains limited, this review primarily focuses on studies in cattle while incorporating selected studies from other species (e.g., rodents and small ruminants, pigs, and humans) to provide mechanistic support. For studies reporting inconsistent findings, the present review weighed the evidence based on study design quality and strength of evidence, giving priority to studies with larger sample sizes, independent animals as the statistical unit, appropriate controls, and properly handled repeated measurements.

## 3. Behavioral Responses of Cattle Induced by Heat Stress

Upon exposure to heat stress, cattle initiate a series of behavioral thermoregulatory responses. These behavioral modifications serve not only as primary adaptive strategies to cope with high ambient temperatures but also as critical macroscopic indicators for assessing the severity of heat stress. The most immediate impact of heat stress is observed in feeding behavior, characterized by significant reductions in both feeding duration and feed intake [11,12]. Under heat stress conditions, cattle exhibit a marked decrease in lying time accompanied by a notable increase in standing time. This postural adjustment possesses distinct physiological significance: standing minimizes the body’s contact area with the ground and maximizes the skin surface area exposed to the air, thereby facilitating heat dissipation via convection and radiation [13,14]. Concurrently, elevated temperatures significantly suppress rumination activity. Given that rumination entails substantial heat production, cattle curtail rumination duration during heat stress to mitigate endogenous heat load [12,15].

Under heat stress conditions, the elevation of respiratory rate and rectal temperature in cattle is among the most conspicuous indicators, and these parameters are also frequently used to identify heat stress in cattle. Under heat stress conditions, the respiratory rate of cattle can surge from a baseline of approximately 30–40 breaths per minute (breaths/min) to as high as 72–89 breaths/min [16]. During periods of severe heat stress, cattle exhibit distinct thermoregulatory behaviors, including tongue protrusion, open-mouth panting, and excessive salivation, which facilitate rapid heat dissipation via evaporative cooling from the oral cavity and respiratory tract [17]. Furthermore, elevated rectal temperature (RT) is a hallmark physiological manifestation of heat stress; upon exposure to thermal load, RT rises significantly from approximately 38.5 °C (at THI > 68) to 39.2 °C (at THI > 88) [18]. It is important to note, however, that all of the aforementioned values were obtained from specific experimental populations and exposure conditions. Consequently, the assessment of heat stress in cattle should be made in conjunction with the intrinsic characteristics of the herd and the surrounding environmental conditions.

## 4. Effects of Heat Stress on Bull Semen Quality

### 4.1. Heat Stress-Induced Decline in Conventional Semen Quality Parameters

Heat stress is a primary environmental factor impairing bull reproductive performance, and its detrimental impact on semen quality has been substantiated by extensive research [19,20]. Regarding sperm motility, the decline induced by heat stress typically exhibits a delayed onset, becoming fully evident in semen parameters approximately two weeks after the onset of heat exposure, though this time frame varies with the exposure protocol (intensity, duration) and the specific endpoint assessed [9,21]. This lag primarily reflects the impact of heat stress on flagellar (sperm tail) formation, which is consistent with the duration of the spermatogenic cycle. Moreover, the detrimental effects of heat stress extend beyond fresh semen to severely compromise post-thaw semen quality. Studies have shown that semen collected during periods of heat stress yields significantly lower post-thaw sperm motility (36.8%) compared with that from bulls under non-heat-stress conditions (53.6%), accompanied by increased post-thaw DNA fragmentation and reduced acrosome integrity and plasma membrane integrity [22]. At the level of sperm production, heat stress simultaneously reduces ejaculate volume and sperm concentration, thereby decreasing the total number of spermatozoa per ejaculation. These effects are largely attributable to heat-stress-induced suppression of androgen synthesis, reduced secretion from the accessory sex glands, and indirect effects such as decreased feed intake and disruption of body water balance [19,23]. It must be emphasized that the magnitude of these effects varies considerably with breed, individual, and rearing environment. Indeed, Netherton et al., based on large-scale ejaculation data, clearly demonstrated that bulls exhibit pronounced individual heterogeneity in their response to heat stress, with distinct “heat-sensitive” and “heat-tolerant” phenotypes [6]. Consequently, the extent to which heat stress affects sperm motility is not a fixed value; it should be evaluated in an integrative manner, taking into account the specific population (breed, age) and the conditions of heat exposure (THI level, duration).

In summary, heat stress poses a multifaceted threat to the reproductive performance of breeding bulls by reducing sperm motility, sperm concentration, ejaculate volume, and post-thaw semen quality. However, differences across studies are attributable to variation in breed, collection frequency, diet composition, and semen-processing protocols, as well as the confounding effects of repeated measurements and within-individual variation. Furthermore, it should be noted that most studies cited herein involve chronic environmental heat exposure (e.g., seasonal summer heat stress lasting weeks to months), whereas the effects of acute, short-term heat exposure (hours to days) on semen parameters remain less well characterized and may follow distinct temporal patterns. It should be noted that most studies cited in this section assess semen quality parameters (e.g., motility, morphology, concentration, post-thaw viability) rather than confirmed fertility outcomes (e.g., conception rates, calving rates, or field fertility). Semen quality serves as a proxy for fertility but does not directly equate to it.

### 4.2. Heat Stress-Induced Alterations in the Semen Biochemical Microenvironment

Seminal plasma constitutes the immediate microenvironment for spermatozoa, providing not only essential nutrients for basal metabolism but also a diverse array of metabolic intermediates and proteolytic enzymes that play pivotal roles in mitigating stress-induced damage [24]. Sustained heat stress profoundly disrupts the homeostasis of energy metabolites within the seminal plasma. In Mediterranean male buffaloes, for example, summer heat stress significantly elevates the concentrations of fructose, acid phosphatase, and α-glucosidase. Paradoxically, this apparent increase in energy substrates coincides with a marked decline in sperm motility, indicating potential impairments in energy utilization or underlying metabolic dysregulation [25].

The detrimental impact of heat stress primarily stems from cellular-level damage triggered by systemic thermal load. Elevated local testicular temperatures directly induce protein denaturation, leading to the inactivation of critical enzymes and functional impairments of structural proteins, which in turn disrupts normal biochemical cascades. This direct heat-induced testicular injury represents the primary driver of spermatogenic damage. In addition to direct thermal injury, heat stress often reduces feed intake, exacerbating a state of negative energy balance. This indirect metabolic effect is distinct from direct thermal injury and may act through separate pathways—for example, by limiting the availability of nutrients and antioxidants needed to support spermatogenesis and counteract oxidative stress. It is important to note that negative energy balance per se does not directly drive an increase in mitochondrial ATP synthesis. Instead, heat stress can directly impair mitochondrial function by disrupting the electron transport chain (ETC)—as evidenced by decreased expression of ETC complexes and reduced oxygen consumption rate in porcine sertoli cells [26]—thereby promoting electron leakage and elevating reactive oxygen species (ROS) generation. While these mechanistic findings were obtained from in vitro porcine sertoli cells, they are consistent with the well-documented accumulation of ROS and oxidative damage in the seminal plasma of heat-stressed bulls [27,28], where excessive ROS exerts sustained oxidative assault on the sperm plasma membrane, mitochondria, and nuclear chromatin. Meanwhile, hyperthermal conditions suppress the activity of key seminal antioxidant enzymes, including superoxide dismutase (SOD), glutathione peroxidase (GSH-Px), and catalase (CAT). This suppression diminishes ROS scavenging capacity, precipitating a profound oxidative-antioxidant imbalance [29,30]. The resulting ROS accumulation further drives sperm DNA fragmentation and oxidative protein damage. Notably, the exceptionally high PUFA content in spermatozoa confers an intrinsic vulnerability to lipid peroxidation, thereby amplifying the structural and functional deterioration induced by heat stress [27,31,32].

## 5. Impact of Heat Stress on Bovine Semen Quality via Systemic Physiological Responses

### 5.1. Heat Stress Affects Bovine Semen Quality via the Neuroendocrine System

Bovine semen quality is fundamentally dependent on the animal’s neuroendocrine system. Under normal physiological conditions, the bovine thermoregulatory system maintains the testicular temperature at an optimal level, thereby ensuring the proper proliferation, differentiation, and maturation of sperm [33,34]. However, sustained high temperatures induce heat stress that disrupts physiological homeostasis, triggering a cascade of systemic neuroendocrine disorders, reproductive axis imbalances, and damage to the testicular microenvironment. These responses interfere with the entire process of spermatogenesis from upstream regulatory pathways to local spermatogenic tissues, ultimately leading to a significant decline in semen quality [35]. Among these mechanisms, neuroendocrine dysregulation serves as the core upstream pathway mediating heat stress-induced reproductive dysfunction in bulls and is a critical factor driving the deterioration of semen quality [36].

**Figure 1:**
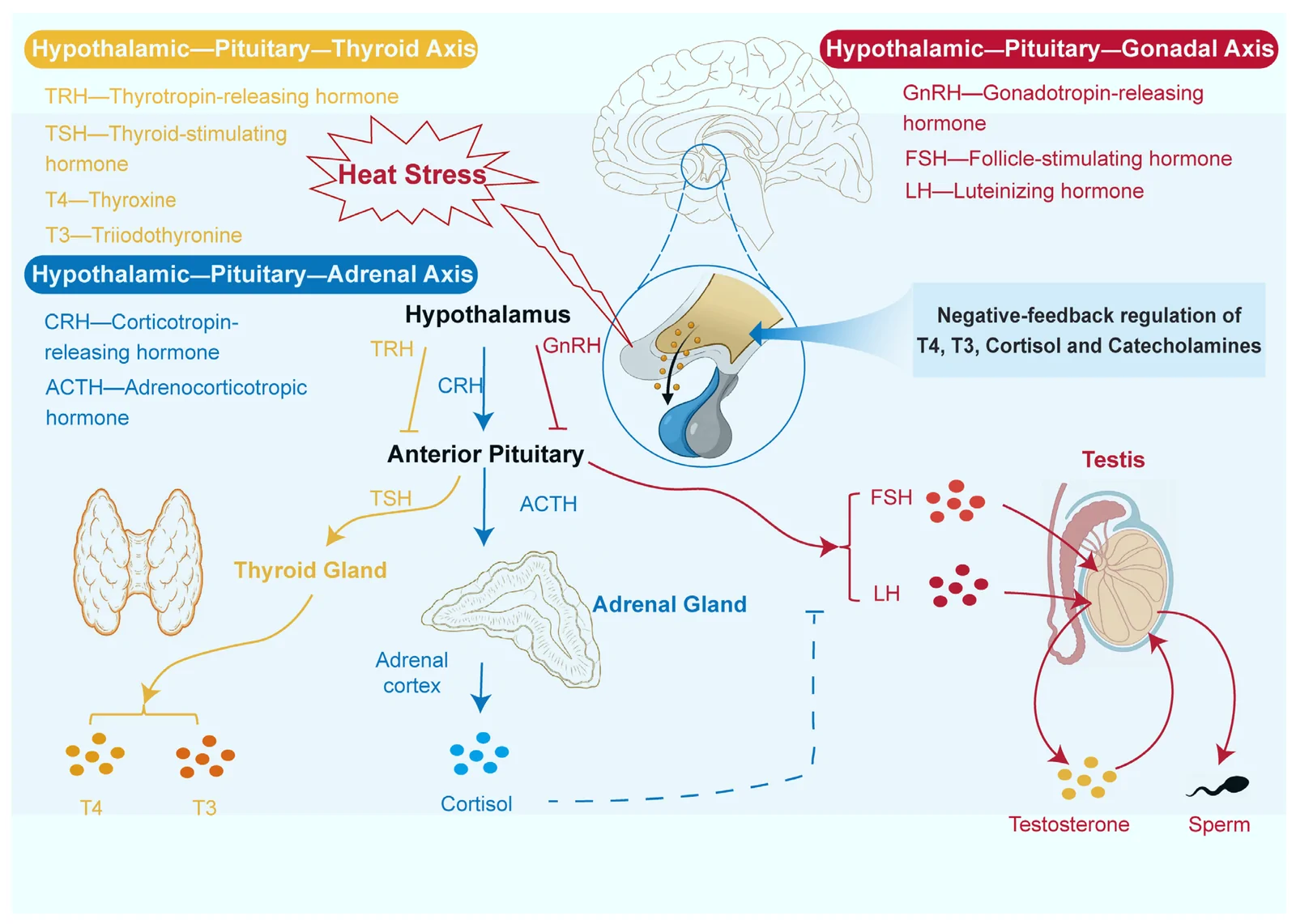
Schematic diagram of the regulatory network of the hypothalamic–pituitary axis and associated endocrine axes under heat stress. The figure shows the regulatory processes of the HPT axis, HPA axis, and HPG axis in response to heat stress, with solid lines indicating established mechanisms and dashed lines indicating mechanisms that remain to be verified. For detailed mechanistic descriptions and supporting references, see Section 5.1 and Section 5.2.

Under heat stress conditions, the function of the hypothalamic–pituitary–thyroid (HPT) axis is significantly suppressed, manifesting primarily as decreased serum concentrations of triiodothyronine (T3) and thyroxine (T4) [37,38], as shown in Figure 1. This adaptive response aids in reducing metabolic heat production and represents a crucial physiological mechanism for maintaining thermal balance in high-temperature environments. Studies indicate that environmental heat suppresses the HPT axis activity in bulls, accompanied by a decline in serum hormone levels such as T3, suggesting that HPT axis suppression may indirectly affect reproductive hormone secretion through complex network interactions [39]. Research has shown that, compared to thermoneutral conditions, heat stress can decrease blood T4 by 45.4%, and T3 by 25.9% [40]. Anjali et al. found that severe heat stress exposure not only reduced T3 and T4 concentrations and elevated cortisol levels but was also associated with a significant decline in serum testosterone. These findings collectively suggest that the suppression of the HPT axis constitutes a pivotal endocrine node in the regulation of reproductive performance under heat stress [38]. However, whether the HPT axis directly modulates male reproduction or merely represents a concomitant phenotype co-regulated with the reproductive axis by upstream stress signals remains difficult to distinguish, and the underlying regulatory mechanisms in bulls require further experimental validation.

The decline in bull reproductive performance under heat stress is closely associated with neuroendocrine system disturbances, with the core mechanism centering on the activation of the hypothalamic–pituitary–adrenal (HPA) axis, as shown in Figure 1. This stress cascade is characterized by the sequential release of corticotropin-releasing hormone (CRH) and adrenocorticotropic hormone (ACTH), culminating in a substantial secretion of glucocorticoids, primarily cortisol. These elevated stress hormones exert significant negative feedback inhibition on the HPG axis [41,42]. In bulls, excessive activation of the HPA axis may also exert a synergistic inhibitory effect on testosterone synthesis via the HPG axis. Research by Anjali et al. demonstrated that the heat stress-induced elevation in blood cortisol levels occurred synchronously with a decline in serum testosterone levels [38]. It should be noted that the synchrony of these hormonal changes currently represents only an observational association and cannot, by itself, establish a synergistic inhibitory interaction between the HPA and HPG axes. Although studies in rodent models have provided preliminary evidence that glucocorticoid-mediated negative feedback can suppress gonadotropin-releasing hormone (GnRH) release and downregulate Luteinizing Hormone (LH) secretion [43], whether this mechanism exerts a similar synergistic effect in bulls remains to be directly verified by experimental evidence. Beyond reproductive function, the activation of the HPA axis also exerts widespread effects on the immune system. On one hand, the released cortisol can impair neutrophil adhesion and migration to inflammatory sites by downregulating the expression of L-selectin on their surface, thereby compromising neutrophil phagocytosis and pathogen clearance efficiency [44]. On the other hand, heat stress can reduce serum immunoglobulin (e.g., IgG) concentrations, further weakening the humoral immune barrier and increasing the animal’s susceptibility to pathogenic microorganisms [45]. Owing to its high sensitivity to heat stress and the relatively well-established detection techniques, plasma cortisol concentration has been widely employed as a reliable biomarker for evaluating the severity of heat stress in cattle in the future. Furthermore, combining cortisol with additional indicators including the THI, body temperature, and respiratory rate into a multi-indicator integrated evaluation system would substantially improve the precision of heat-stress assessment.

In addition to the aforementioned hormones, heat stress induces significant changes in the levels of metabolic-related hormones such as insulin, growth hormone (GH), and leptin, thereby exacerbating spermatogenic impairment and reduced male fertility. Insulin, as a core hormone regulating glucose and lipid metabolism, undergoes marked changes in its secretion under heat stress. Although heat stress induces a negative energy balance by suppressing dry matter intake, in contrast to the reduction in insulin levels typically observed in feed-restricted animals, heat-stressed cattle paradoxically exhibit elevated plasma insulin levels, with an increase of approximately 30–37% [16,46]. Heat stress compromises dry matter intake through appetite suppression, thereby directly impairing productive performance; however, this simultaneously triggers a cascade of adaptive endocrine responses in the organism. By maintaining a state of elevated plasma insulin coexisting with relatively low blood glucose, the body suppresses adipose tissue mobilization and redirects the allocation of substrates such as glucose, thereby reducing metabolic heat production and preserving thermal homeostasis, while concurrently safeguarding energy supply and meeting production demands. GH is a critical endocrine factor regulating growth, protein synthesis, and thermogenesis, and substantial evidence indicates that heat stress inhibits its secretion [37,47]. Mitra et al. observed that plasma GH concentration in cattle under heat stress at 35 °C (13.5 ng/mL) was significantly lower than that in the thermal neutral zone at 18 °C (18.2 ng/mL) [48]. The physiological significance of downregulated GH levels may lie in the fact that GH possesses a high thermogenic potential; thus, its reduced secretion helps lower metabolic heat production, thereby facilitating thermal balance. Furthermore, the decline in GH directly leads to reduced hepatic synthesis of insulin-like growth factor-I (IGF-I) [37]. Rhoads et al. found that IGF-I concentrations could decrease by 16% under heat stress. Given the critical regulatory role of IGF-I in promoting the proliferation and differentiation of testicular Sertoli cells and spermatogenesis, its downregulation likely constitutes one of the important pathways through which heat stress impairs male reproductive function [47]. Collectively, heat stress suppresses the GH/IGF-I axis, which, while prioritizing thermal homeostasis, curtails certain growth and anabolic processes and indirectly perturbs the testicular microenvironment. This endocrine pathway therefore constitutes one of the key mechanisms by which heat stress compromises semen quality in bulls. Leptin, an adipokine primarily secreted by white adipocytes, serves as a key indicator of energy reserves and appetite suppression [49]. Previous studies have demonstrated that summer heat stress can lead to elevated leptin levels in cattle [50,51], a process potentially governed by multiple mechanisms, including adipokine resistance, inflammatory signaling, and adipose tissue remodeling. At the level of male reproduction, a pathological elevation in leptin levels can markedly suppress spermatogenesis [52]. Mechanistically, this suppression is primarily mediated by the direct action of leptin on Leydig cells, where it downregulates the steroidogenic pathway and thereby inhibits testosterone synthesis, while simultaneously impairing the metabolic support function of Sertoli cells, consequently disrupting the process of spermatogenesis [53,54,55]. Collectively, heat stress elevates serum leptin levels and directly targets testicular cells to suppress spermatogenesis, thereby constituting one of the key endocrine pathways through which heat stress compromises the semen quality of bulls.

### 5.2. Direct Inhibitory Effects of HPG Axis Dysregulation on Testicular Microenvironment and Testosterone Synthesis

The HPG axis serves as the central hub for maintaining male reproductive function and spermatogenic homeostasis [56]. Heat stress severely disrupts the homeostasis and physiological function of the HPG axis, ultimately leading to reproductive impairment. The primary site of action lies at the central level, and this process is illustrated in Figure 1: elevated temperatures directly inhibit the synthesis and secretion of GnRH in the hypothalamus, which subsequently impedes the production and release of LH and follicle-stimulating hormone (FSH) from the anterior pituitary [57]. As the core gonadotropins regulating male spermatogenesis, FSH primarily targets testicular Sertoli cells, modulating their nutritional and phagocytic functions to provide essential microenvironmental support for the differentiation and maturation of spermatogenic cells. Meanwhile, LH activates receptors on Leydig cell membranes to initiate and sustain the cascade of testosterone biosynthesis [58,59]. Notably, the dysfunctions of the testes and the endocrine centers are not independent events but rather constitute a multi-level, coordinated impairment orchestrated through the HPG axis. Under heat stress, the concurrent decline in serum LH and FSH concentrations simultaneously compromises FSH-dependent trophic support of Sertoli cells and attenuates LH-driven testosterone biosynthesis in Leydig cells, and the synergistic perturbation of these two compartments collectively disrupts the testicular microenvironment essential for spermatogenic cell survival and differentiation. Consistent with this mechanistic framework, numerous studies have demonstrated that summer heat stress significantly reduces serum GnRH, LH, and FSH levels in cattle relative to thermoneutral seasons, and that these hormonal disturbances exhibit a marked temporal association with declining semen quality and testicular dysfunction [60,61]. Collectively, this evidence establishes the HPG axis as a central driver of heat stress-induced reproductive impairment and subfertility, thereby underscoring its role as a critical therapeutic target for reproductive regulation in male animals and for the development of intervention strategies against heat stress-related reproductive damage.

### 5.3. Molecular Regulation of Semen Quality by Heat Stress-Related Gene Expression

Oxidative stress induced by heat stress and its extensive damage to the reproductive system serve as the pivotal axis linking these mechanisms. This molecular regulatory framework encompasses heat shock protein (HSP) responses and the cascading activation of apoptotic pathways. Collectively, these factors target the testicular spermatogenic microenvironment, ultimately resulting in decreased sperm motility, morphological abnormalities, and compromised DNA integrity.

HSPs are considered molecular biomarkers for evaluating the severity of heat stress and represent the primary molecular defense line against heat-induced injury. When ambient temperatures exceed physiological thresholds, the reactive oxygen species (ROS) generated by oxidative stress lead to the massive accumulation of intracellular denatured proteins. This triggers the activation of heat shock transcription factors, which in turn initiate the rapid transcription of HSP genes to protect cells from oxidative damage [9]. Research indicates that under heat stress, the expression of core subtypes such as HSP70 and HSP90 is significantly upregulated in testicular tissues. These upregulated HSPs mitigate acute damage to spermatogenic cells by facilitating the repair of denatured proteins, scavenging oxidative damage products, and maintaining cellular structural integrity. However, sustained high-intensity heat stress disrupts this compensatory equilibrium; excessive accumulation of HSPs can negatively modulate cellular signaling pathways, thereby inhibiting the normal proliferation and differentiation of spermatogenic cells [62]. Moreover, when ROS accumulation surpasses the antioxidant capacity of the organism, it induces mitochondrial dysfunction and activates caspase-mediated apoptotic pathways, triggering the programmed death of testicular spermatogonia and spermatocytes [63]. Solanki et al. found that HSP70 expression in the pre-freezing semen of Gir bulls was positively correlated with sperm motility, viability, acrosome integrity, and fertilizing capacity, pointing to HSP70 as a reliable marker of seminal heat tolerance and fertility in this breed [64]. Four HSP70 genes are characterized in cattle, HSP70-1, HSP70-2, HSP70-3, and HSP70-4, among which HSP70-2 participates in spermatogenesis and protects cells against apoptosis and oxidative stress [65]. Given these gene- and breed-specific properties, HSPs, and HSP70-2 in particular, may serve not only as biomarkers of heat tolerance and semen quality but also as candidate targets for mitigating heat-stress-induced reproductive damage.

### 5.4. Impact of Heat Stress on Spermatogenesis via Epigenomic Remodeling

Spermatogenesis is a highly orchestrated and dynamic process that is particularly susceptible to environmental perturbations. Heat stress can induce extensive epigenomic remodeling in germ cells. Although such remodeling does not alter the DNA sequence per se, it can profoundly affect spermatogenic efficiency and functional integrity by modulating transcriptional activity, maintaining genomic stability, and mediating gamete interactions. During spermatogenesis, which is accompanied by epigenetic reprogramming, heat stress often leads to aberrant deposition of sperm epigenetic marks by disrupting the homeostasis of non-coding RNAs, interfering with the normal establishment of DNA methylation patterns, and disturbing the dynamic equilibrium of RNA chemical modifications. This section comprehensively explores the epigenetic mechanisms by which heat stress affects spermatogenesis from three dimensions: non-coding RNA regulation, DNA methylation remodeling, and m^6^A methylation modification.

#### 5.4.1. Impact of Heat Stress on Spermatogenesis via Non-Coding RNAs

Non-coding RNAs (ncRNAs), primarily including microRNAs (miRNAs), long non-coding RNAs (lncRNAs), and circular RNAs (circRNAs), have attracted considerable attention as key regulators of male reproductive function. Among these, miRNAs constitute the most extensively characterized class of ncRNAs and act as pivotal post-transcriptional regulators in animals, playing indispensable roles in modulating testicular function and semen quality [66,67,68]. Heat stress has been shown to substantially alter the expression profiles of more than 300 miRNAs in bovine spermatozoa. Notably, miR-181d is enriched in spermatozoa following exposure to elevated temperatures, where it disrupts cellular physiological architecture and ultimately compromises fertilization capacity and embryonic developmental competence [69]. Furthermore, scrotal heat stress impairs testicular and epididymal function through the downregulation of multiple miRNAs—including miR-9-5p, miR-34b, miR-146a, and miR-200b—in bovine seminal extracellular vesicles and spermatozoa. This heat stress-induced miRNA dysregulation perturbs the regulatory networks governing genes involved in cell proliferation and differentiation, thereby exacerbating reproductive dysfunction [70].

circRNAs are abundantly expressed in bovine testicular tissue, largely owing to their covalently closed loop structure, which confers exceptional stability against exonuclease-mediated degradation [71,72]. circRNA has been demonstrated to act as a molecular sponge that sequesters and isolates miRNAs, thereby relieving the inhibitory effects of miRNAs on their target genes; however, such “circRNA–miRNA–mRNA” interactions are largely predicted at the level of regulatory networks [73,74]. Heat stress has been shown to alter the circRNA expression profile in mouse testes, and it has been speculated that this may impair spermatogenesis by perturbing the “circRNA–miRNA–mRNA” regulatory network [75]. Nevertheless, circRNA dynamics in bovine testicular tissue under heat stress remain largely unexplored, and the biological functions and regulatory mechanisms of specific circRNAs in heat stress-induced impairment of male fertility await experimental validation.

LncRNAs are transcripts exceeding 200 nucleotides in length that possess no or extremely limited protein-coding capacity. They can participate in the post-transcriptional regulation of gene expression through interactions with molecules such as miRNAs or proteins. Studies have shown that lncRNA *AK015322* is highly expressed in human spermatogonial stem cells and regulates their proliferation by acting as a sponge for miR-19b-3p [76]. The lncRNA *Mrhl* influences mouse spermatogenesis through its interaction with the p68 protein [77]. In addition, *LNC_003902* and *LNC_007258* are involved in regulating porcine testicular development and spermatogenesis by modulating *GATA4* and *NKAP*, respectively [78]. Research on lncRNAs in the bovine reproductive system remains limited, although accumulating evidence points to their potential roles in testicular development and spermatogenesis [79]. Notably, heat stress markedly upregulates lncRNA *4930555K19Rik* and the transposable element *LINE-1* in male germ cells, which impairs reproductive function [80]. These findings provide a valuable framework for dissecting the molecular mechanisms by which lncRNAs contribute to heat stress-induced bull reproductive impairment.

Taken together, non-coding RNAs constitute a pivotal epigenetic dimension through which heat stress perturbs spermatogenesis, with their influence extending across multiple layers of post-transcriptional regulation. Collectively, they form an integrated, multi-layered regulatory network that underpins the aberrant deposition of epigenetic marks in spermatozoa under heat stress, thereby providing a mechanistic framework for understanding and, ultimately, mitigating heat-stress-induced reproductive impairment.

#### 5.4.2. Impact of Heat Stress on Spermatogenesis via DNA Methylation

DNA methylation, an epigenetic modification that adds a methyl group to the fifth carbon of cytosine within CpG dinucleotides (5-methylcytosine, 5mC), is essential for genomic imprinting, gene expression regulation, chromosomal inactivation, and reproductive development [36]. Emerging evidence preliminarily suggests that DNA methylation may be involved in mediating the effects of heat stress on semen quality in cattle. Kumar et al., using whole-genome DNA methylation sequencing, revealed that heat stress induced differential methylation patterns in water buffalo sperm, and these patterns were associated with the decline in semen quality [81]. It should be noted that the current evidence only reveals a correlation between methylation alterations and the decline in semen traits, and cannot yet directly establish that DNA methylation is a causal mediator of heat stress-induced impairment of spermatogenesis. This is primarily because heat stress can concurrently damage sperm through multiple pathways, including oxidative stress and hormonal disturbances, and the observed methylation changes may represent a concomitant phenomenon of these injuries rather than the primary cause. Therefore, direct validation through functional experiments—such as methyltransferase inhibitors and site-specific epigenome editing—is still required. Given its potential role in spermatogenesis, DNA methylation markers are expected to be incorporated into the selection programs for thermotolerant breeding bulls, thereby mitigating the adverse effects of global warming on livestock production [82]. However, the stability, specificity, and feasibility of large-scale detection of these biomarkers remain to be validated, and further research is needed to progressively advance from observational associations toward causal mechanistic verification.

#### 5.4.3. Impact of Heat Stress on Spermatogenesis via m^6^A Methylation

Similar to DNA methylation, mRNA is subject to multiple types of methylation modifications, including m^6^A, m^5^C, and m^7^G. Among these, m^6^A (N6-methyladenosine) methylation is one of the most abundant modifications in mammalian mRNA and plays a pivotal role throughout the entire process of spermatogenesis, encompassing mitosis, meiosis, and spermiogenesis [83]. The dynamic regulation of m^6^A modification is coordinated by three classes of functional proteins: methyltransferases (“writers”) that establish m^6^A sites, demethylases (“erasers”) that mediate reversible removal, and reader proteins that specifically recognize m^6^A marks and recruit downstream effector factors. Together, these components constitute a finely tuned regulatory network governing multiple stages of spermatogenesis, including RNA splicing, nuclear export, stability, translation, and degradation [84]. Heat stress has been reported to disrupt the homeostasis of the HPG axis via the FTO/IGF2BP2/m^6^A-Anxa2 pathway, resulting in reduced sperm motility and thereby highlighting the critical regulatory role of m^6^A methylation in heat stress-induced male reproductive impairment [85]. In a related study using boar Sertoli cells, heat stress upregulated the methyltransferase METTL3 and the reader protein YTHDF1, enhancing the m^6^A modification of SOS2 mRNA and promoting its translation. This in turn activates the ERK1/2 signaling pathway and synergistically promotes NLRP3 inflammasome activation, jointly mediating cellular injury [86]. Collectively, these findings indicate that m^6^A methylation exerts a crucial regulatory influence on heat stress-induced reproductive damage in males. However, current evidence on m^6^A methylation in heat stress-induced reproductive damage in bulls remains fragmented, and the underlying pathways have yet to be systematically delineated. Its potential as a marker for heat tolerance evaluation or as an interventional target therefore still awaits mechanistic elucidation and phenotypic validation.

## 6. Impact of Heat Stress on Semen Traits via the “Gut–Testis” Axis

The gut harbors a complex microbial ecosystem that interacts intimately with the host and profoundly shapes host health and environmental adaptability. This microbial community, often termed the host’s “second genome,” plays an indispensable role in intestinal development, nutrient digestion and utilization, and immune regulation [87]. The gastrointestinal tract harbors a taxonomically rich and numerically abundant microbiota, and in ruminants this community reaches its highest density within the rumen [88]. Through diverse and intricate signaling mechanisms, the gut microbiome communicates with distal host organs—including the brain, liver, muscle, and reproductive system—thereby constituting an indispensable determinant of animal health. Heat stress, a pervasive challenge in the livestock industry, exerts dual effects on fertility: it directly damages spermatogenesis while concurrently impairing reproductive performance via disruption of gut microbial homeostasis. Thus, delineating the mechanistic pathways through which heat stress influences male reproduction along the “gut–testis” axis is of considerable theoretical and practical importance for the reproductive management of breeding bulls.

### 6.1. Gut Microbiota as a Mechanistic Basis for the Regulation of Male Reproduction

In recent years, the influence of the gut microbiota on male reproductive capacity has attracted increasing attention, and the “gut–testis” axis has consequently become a research hotspot in the field of reproductive biology [89,90]. A growing body of reviews has delineated the mechanisms by which the gut microbiota modulates spermatogenesis and sex steroid hormone synthesis through pathways including immunomodulation, metabolism, and signal transduction [91,92], thereby establishing a foundation for a deeper understanding of the impact of the gut microbiota on male reproduction and for the development of subsequent therapeutic strategies.

#### 6.1.1. Intestinal Barrier and Immune Signaling: The Structural Basis of the “Gut–Testis” Axis

The intestinal barrier serves as the structural foundation for the functional integrity of the “gut–testis” axis, and its stability is closely associated with gut microbiota homeostasis [93,94]. Dysbiosis leads to increased intestinal barrier permeability, allowing endotoxins such as lipopolysaccharide (LPS) to enter the systemic circulation via the portal vein or lymphatic system, thereby reducing serum testosterone levels and constituting an important pathological factor in hypogonadism [95]. At the molecular level, LPS can bind to Toll-like receptor 4 (TLR4) expressed on the surface of Leydig cells and Sertoli cells, activating the TLR4/MyD88/NF-κB signaling pathway, which triggers inflammatory responses and impairs testicular function [96,97]. Meanwhile, the gut-associated lymphoid tissue can recognize microorganisms and secrete cytokines such as IL-6, TNF-α, and TGF-β, which reach the testis through the bloodstream to modulate the local immune microenvironment and influence the balance between spermatogenic cell proliferation and apoptosis [10,93]. Conversely, testicular immune cells can also feedback-regulate intestinal immune homeostasis, thereby establishing a bidirectional communication pathway. Collectively, the synergy between barrier integrity and immune signaling constitutes the initial cascade through which the microbiota influences reproduction.

#### 6.1.2. Metabolic Signaling: Short-Chain Fatty Acids and Microbiota-Derived Metabolites

Microbial fermentation of dietary fiber in the gut yields short-chain fatty acids (SCFAs), including acetate, propionate, and butyrate. Beyond serving as an energy source for intestinal epithelial cells, these molecules can also act as signaling mediators that enter the circulation and exert distal regulatory effects. In pigs, Guo et al. demonstrated that *Muribaculaceae* is positively associated with semen quality, an effect attributed to the alleviation of inflammation via SCFAs, thereby improving seminal parameters [98]. Conversely, high-energy diets induce gut microbiota dysbiosis in sheep, characterized by reduced abundance of *Ruminococcaceae_NK4A214*, which perturbs vitamin A metabolism, triggers epididymal inflammation and aberrant testicular gene expression, and consequently impairs spermatogenesis [89]. Furthermore, the microbiome-derived metabolite 3-hydroxyphenylacetic acid has been shown to promote spermatogenesis in aged mice by upregulating GPX4 to suppress ferroptosis [99]. Collectively, these findings indicate that microbial metabolism constitutes a central link in the functional regulation of the “gut–testis” axis, and these microbe-derived metabolites may serve as potential approaches to improve male fertility.

#### 6.1.3. Other Signal Transduction: Endocrine and Exosome-Mediated Long-Distance Regulation

At the level of signal transduction, gut microbiota may modulate nitric oxide (NO) production through arginine metabolism, and the physiological levels of NO are critical for male reproduction [10,100,101]. In addition, neurotransmitters synthesized by gut microbiota, such as serotonin and γ-aminobutyric acid, may also participate in modulating the functional activity of the HPG axis, thereby further mediating the signaling crosstalk between the gut and the reproductive system [102,103]. Gut microbiota can also influence semen traits through exosomal miRNAs. Li et al. demonstrated that gut microbiota dysbiosis leads to aberrant upregulation of miR-211-5p in intestinal exosomes, which suppresses the expression of coiled-coil domain-containing meiosis-specific proteins in the testis, disrupts the meiotic process, and thereby impairs spermatogenesis, establishing exosomal miRNAs as core signaling mediators through which gut microbiota dysbiosis compromises male reproductive function [104]. Chen et al. further reported that dysbiosis alters the overall function of circulating exosomes, leading to aberrant retinoic acid metabolism in the testis and consequently impairing meiosis and spermatogenesis [105]. Conversely, fructo-oligosaccharides can regulate the expression of miR-690 in intestinal epithelial exosomes, increase the abundance of the beneficial bacterium *Eubacterium*, ameliorate microbial dysbiosis, and thereby protect testicular function [106]. Collectively, exosome-mediated miRNA signaling constitutes an important pathway through which the microbiota remotely regulate the spermatogenic microenvironment of the testis.

#### 6.1.4. Protective Effects of Probiotics on Reproductive Function

Accumulating evidence suggests that specific probiotics improve semen quality through multiple pathways, including antioxidant, anti-inflammatory, and neuroendocrine regulation. At the antioxidative level, probiotics can enhance seminal antioxidant capacity by producing enzymes such as SOD and GSH-Px, thereby attenuating ROS-induced damage. In mice, *Lactobacillus rhamnosus* not only improved sperm count and motility but also preserved sperm ultrastructure, DNA integrity, and chromatin condensation status [107]. Two antioxidant probiotic strains—*Lactobacillus rhamnosus CECT8361* and *Bifidobacterium longum CECT7347*—have been shown to improve both ejaculate volume and sperm motility in azoospermic patients [108]. Likewise, *Bacillus amyloliquefaciens TOA5001* enhanced semen quality in roosters through its antioxidant activity [109]. At the anti-inflammatory level, *Enterococcus faecium* and *Clostridium butyricum* protect spermatogenesis and increase the number of mature spermatozoa in the cauda epididymis by remodeling the gut microbiota to reduce LPS production induced by systemic heat stress [110]. In addition, probiotics can also influence hormone secretion levels. Feeding *Lactobacillus reuteri* to aged mice promotes testicular cell proliferation and elevates serum testosterone levels [111]. *Bifidobacterium alleviates* the ciprofloxacin-induced decrease in serum testosterone levels in mice through its antioxidant and anti-inflammatory effects, while also increasing the number of Leydig cells and testosterone synthesis [112]. To date, the reproductive protective effects of the aforementioned probiotics have been validated in various animal models and limited human studies, laying a solid foundation for their future application in the treatment of male reproductive disorders. However, as shown in Table 1, the available evidence is heterogeneous in terms of model, dose, route, and duration, and direct evidence for the reproductive benefits of probiotics in breeding bulls remains scarce. Further elucidation of the key strains, molecular targets, and the regulatory mechanisms underlying the “gut–testis” axis is warranted to advance their clinical and agricultural application.

#### 6.1.5. Impact of Rumen Microbiota on Male Reproduction in Ruminants

In ruminants, the rumen harbors a substantially greater microbial density and diversity than the intestinal tract of monogastric animals, enabling the efficient digestion of dietary fiber and providing the host with a primary source of energy. In monogastric species, microbial metabolism is largely confined to the intestine, and the resultant metabolites enter the systemic circulation via the portal vein after passing through the liver, yielding low production and low utilization efficiency. In contrast, microbial fermentation in ruminants is centered on the rumen, and the volatile fatty acids (VFAs) generated—predominantly acetate, propionate, and butyrate—can account for more than 70% of the host’s total energy intake. These VFAs thereby directly modulate systemic energy metabolism and supply both substrates and energy for spermatogenesis and steroidogenesis [113,114].

In recent years, accumulating evidence indicates that rumen microbiota participate in regulating reproductive function in ruminants through their metabolites, with SCFA-mediated signaling, particularly that of butyrate, being of central importance. For instance, polystyrene microplastics have been shown to impair spermatogenesis in sheep by disrupting rumen microbiota-derived butyrate signaling [115]. At the level of nutritional intervention, dietary supplementation with N-acetylcysteine enhances sperm antioxidant status, energy supply, and lipid homeostasis by optimizing the rumen microbiome and its metabolic axis in goats, thereby improving overall semen quality [116]. Similarly, plant-derived extracts such as the ethanolic extract of *Moringa oleifera* leaves and can reshape the rumen microbiome and metabolome, upregulating endocrine indicators including testosterone and dehydroepiandrosterone while enhancing sperm motility [117]. Notably, a study on Bohai Black cattle reported that dietary supplementation with *Bacillus maritimus N2* remodeled the rumen microbiome–host metabolic axis and upregulated testicular molecular signatures associated with reproduction [118], thereby providing supportive evidence for the feasibility of probiotics improving male fertility through the rumen microbiota and strengthening the case for the rumen–testis axis.

**Figure 2:**
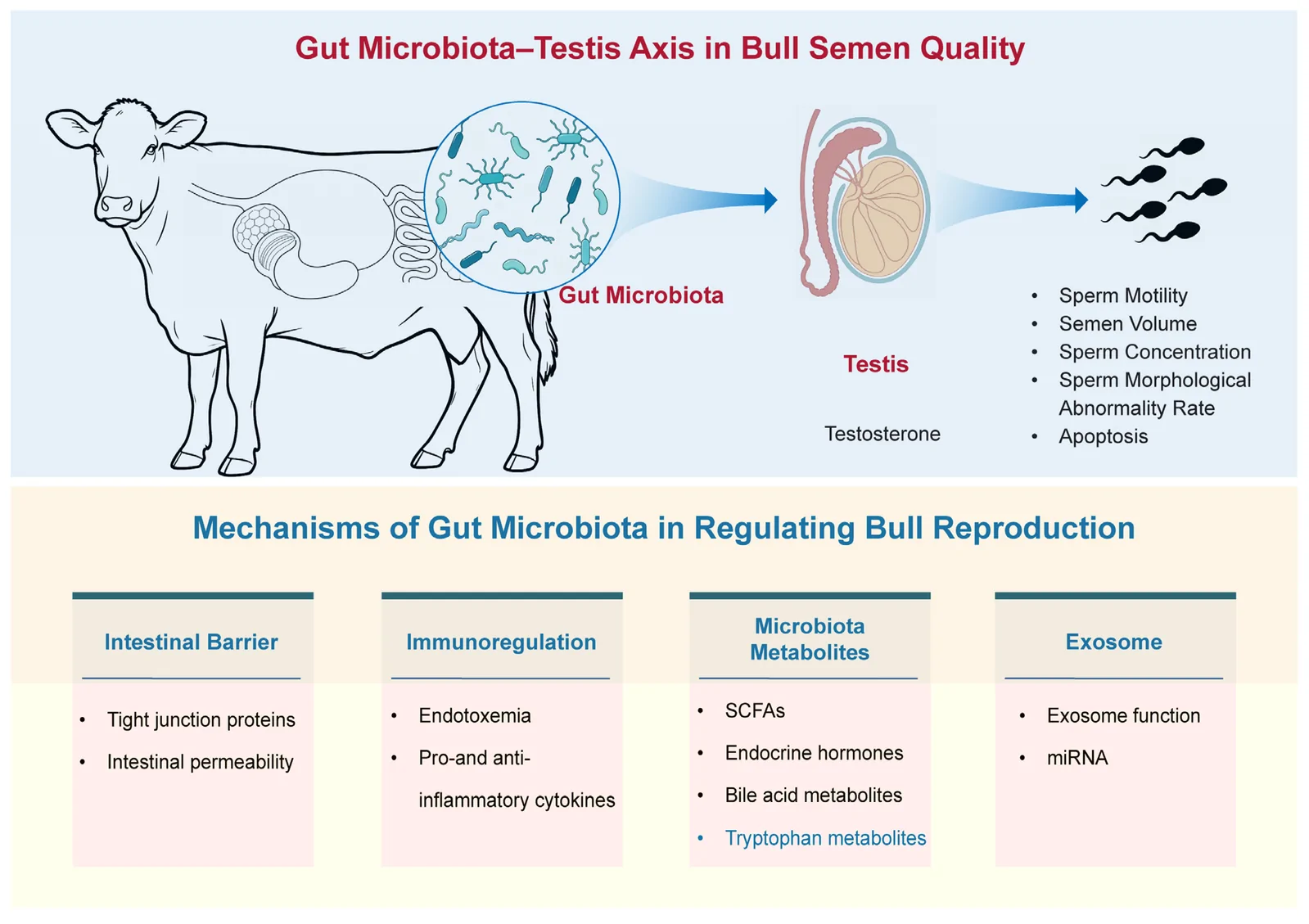
Schematic diagram of the “gut–testis” axis in regulating bull semen quality. Upper panel illustrates the potential pathways through which gut microbiota influence testicular function and semen quality in bulls; the lower panel summarizes the underlying mechanisms. Among these, only bile acid metabolism is supported by direct evidence in bulls, whereas the remaining mechanisms—extrapolated from other animal models—require further validation in bulls. For detailed mechanistic descriptions and supporting references, see Section 6.1.

Collectively, these findings suggest the potential of rumen microbiota in ruminants to influence spermatogenesis and testosterone synthesis through fermentation-derived metabolites—particularly VFAs—and the host–microbial metabolic axis they mediate, and point to promising applications for enhancing bull fertility through targeted modulation of the rumen microbiome. Figure 2 is a schematic diagram illustrating the pathways and mechanisms through which the “gut–testis” axis regulates bull semen quality. However, studies on cattle remain limited, and the proposed regulatory mechanisms are largely extrapolated from other species. Future research should further validate the mechanisms by which the microbiota regulates bull semen quality, with the aim of laying a foundation for enhancing the reproductive potential of bulls.

### 6.2. Heat Stress Impairs Semen Traits via the “Gut–Testis” Axis

Heat stress is one of the major environmental factors responsible for the decline in male reproductive performance. Beyond its direct effects on the testis, it also compromises semen quality through the “gut–testis” axis. However, direct evidence in bulls remains limited at present. Only He et al. observed that heat-stressed bulls showed reduced abundance of *Akkermansia muciniphila* in feces and poorer semen quality, and that transplanting their fecal microbiota into mice decreased testicular germ cells and epididymal spermatozoa. Correlation analysis between the gut microbiota and metabolites of the bulls suggested that *Akkermansia muciniphila* regulates secondary bile acid metabolism and intestinal retinol absorption, thereby modulating testicular retinoic acid levels and mitigating heat stress-induced semen decline [119]. This provides the first direct evidence elucidating the “gut–testis” axis signaling pathway in cattle.

In closely related ruminant species, subsequent studies have further reinforced this mechanism. Heat stress-induced gut microbial dysbiosis can trigger excessive arachidonic acid synthesis in male goats, inducing testicular inflammation and spermatogenic arrest; conversely, melatonin can restore sperm motility by repairing gut microbiota homeostasis and suppressing this metabolic pathway [120]. This further indicates that the metabolic output of the gut microbiota plays a pivotal role in heat stress-induced reproductive damage.

In monogastric animals, heat stress-induced gut dysbiosis and barrier impairment promote the translocation of LPS into the circulation, triggering cascades of testicular inflammation and oxidative stress [121]; L-arginine can alleviate inflammation and protect the spermatogenic microenvironment by modulating the ratio of beneficial to harmful gut bacteria [122]; and suppression of *Lachnospiraceae_XPB1014* abundance elevates serum LPS levels, thereby impairing semen quality and testosterone secretion [123]. The consistent reproduction of these mechanisms across other mammalian species suggests a degree of cross-species conservation.

However, ruminants and monogastric animals differ fundamentally in gastrointestinal architecture, fermentation patterns, and microbial composition. Therefore, extrapolating the above mechanisms to bulls warrants caution. Future research should prioritize the validation of core pathways—such as LPS translocation and secondary bile acid metabolism—directly in bulls, in order to provide direct and reliable evidence for breeding management in sires.

## 7. Potential Intervention Strategies to Mitigate Heat Stress-Induced Damage

### 7.1. Establishment of a Multi-Omics Integration and Comprehensive Heat Tolerance Evaluation Model for Bulls

Current studies on the effects of heat stress on bull semen quality are largely restricted to single-omics approaches, which cannot fully capture the systemic features of heat stress-induced damage. Nevertheless, recent omics-based investigations conducted in bulls have preliminarily demonstrated the feasibility of multi-omics integration. Specifically, Guo et al. combined proteomics with metabolomics to demonstrate that spermine enhances the cryotolerance of bull sperm via antioxidant pathways [24]. Talluri et al. integrated transcriptomic, proteomic, and metabolomic datasets to systematically identify the core molecules and key pathways governing sperm metabolism that influence fertility in bulls [124]. Furthermore, Li et al. integrated the sperm cell transcriptome with the seminal plasma metabolome in Holstein stud bulls, thereby dissecting the molecular regulatory mechanisms underlying sperm motility from the cross-level perspective of “gene expression–metabolite” [125].

Collectively, these examples demonstrate that multi-omics integrative analysis of bull semen is technically feasible and can yield systemic information that is unattainable through any single-omics approach. However, the existing bull multi-omics studies have predominantly focused on fertility and cryotolerance evaluation, whereas multi-omics-integrated analyses specifically targeting spermatogenesis under heat-stress conditions remain virtually unexplored. A more integrated strategy is therefore warranted, one that combines genomic, transcriptomic, epigenomic, proteomic, and microbiome data to construct a hierarchical molecular regulatory network underlying spermatogenesis under thermal stress. In parallel, single-cell omics technologies offer the resolution needed to dissect how heat stress perturbs individual spermatogenic lineages, thereby pinpointing cell-type-specific responses and identifying key regulatory nodes.

Building on the integration of multi-omics data, an attempt can be made to establish a multi-dimensional evaluation model that integrates genetic markers, epigenetic markers, and gut microbiota features. It should be emphasized that the above molecular and microbial indicators are still at the candidate marker stage, and whether they can become effective validated predictors still needs to be confirmed through large-scale cohort validation and comparison with traditional semen quality indicators. Furthermore, the assessment of semen quality in heat-stressed breeding bulls could be adopted as the primary objective: by combining candidate markers with validated physiological indicators, dynamic monitoring and early warning of changes in semen quality of heat-stressed bulls can be achieved, thereby providing a basis for proactive intervention and enhancing the precision of breeding bull management.

### 7.2. Establishment and Breeding Application of Heat Stress Tolerance Intervention Strategies

Currently, the primary heat-stress management strategy for cattle is cooling-based environmental management. Physical cooling measures—such as installing shade structures, configuring combined sprinkler–fan systems, and optimizing barn ventilation—can directly reduce the THI, thereby alleviating both the reduced feed intake and the thermoregulatory burden in breeding bulls. Although environmental cooling can help maintain production performance in the short term, its effects are largely external and transient, and cannot be internalized as a durable, individual-level heat tolerance in bulls. Nor can it fully block the deep-seated damage that heat stress inflicts on spermatogenesis. To date, although high-throughput sequencing technologies have identified numerous candidate genes, metabolites, and gut microbial markers associated with heat-stress responses and semen quality, the translation of these findings into practical application in breeding-bull production remains very limited. Bridging the gap between these molecular and microbial discoveries and actionable heat stress tolerance interventions is therefore a pressing challenge. Therefore, nutritional intervention and molecular breeding collectively constitute the two fundamental strategies for mitigating heat stress.

#### 7.2.1. Precise Nutritional Intervention via Dietary Supplementation

Heat stress induces the accumulation of reactive oxygen species (ROS) within the body, which in turn impairs spermatogenesis and constitutes one of the primary causes of the decline in semen quality in bulls. Consequently, dietary supplementation with antioxidant feed additives represents an effective strategy for alleviating heat stress-induced deterioration of semen quality. Selenium and vitamin E, functioning as essential constituents and synergistic cofactors of key enzyme systems such as glutathione peroxidase, scavenge excess ROS and repair lipid peroxidation damage to the sperm membrane, thereby safeguarding the structural integrity of spermatozoa [126,127]. Dietary melatonin supplementation has likewise been demonstrated to mitigate the adverse effects of heat stress on buffalo semen quality by directly scavenging free radicals and restoring redox homeostasis [128]. At the level of lipid metabolism, ω-3 polyunsaturated fatty acids (e.g., docosahexaenoic acid) improve the phospholipid composition of the sperm membrane and enhance membrane fluidity, thereby increasing sperm motility and viability in Holstein bulls under heat stress conditions [28]. In addition, certain metabolites secreted by probiotics also exert antioxidant effects; notably, Bacillus maritimus has been shown to improve the testicular molecular signatures of Bohai Black cattle by remodeling the rumen microbiome–host metabolic axis [118]. Although direct evidence that probiotics alleviate heat stress-induced declines in bull semen quality remains limited, findings from monogastric animal studies may nonetheless provide a foundation for developing individualized probiotic intervention regimens.

#### 7.2.2. Molecular Breeding Improvement Based on Gene Editing

Beyond nutritional intervention, functional validation and genetic improvement of candidate heat stress genes represent another key avenue toward heritable enhancement of thermotolerance across cattle herds. Using gene-editing platforms such as CRISPR to generate conditional knockout or knock-in animal models enables verification of the functional necessity of core molecules in heat stress-induced reproductive damage at the whole-animal level. For biomarkers whose functions have been firmly established, they should be further incorporated into the selection schemes for thermotolerant breeding bulls, thereby systematically strengthening herd-level thermotolerance from a genetic perspective. Notably, gene editing applications in heat-tolerant cattle breeding to date have primarily focused on thermotolerance itself as the phenotypic target, whereas semen quality under heat stress has not yet been directly employed as a primary evaluation endpoint. For example, Song et al. established a novel precision breeding system that achieves non-invasive editing of fertilized eggs through a “one-step” strategy, successfully generating SLICK gene-edited cattle with enhanced heat tolerance [129]. The bull possesses typical smooth, short-haired coats, which primarily improves coat structure and thermoregulatory capacity. Meanwhile, Cuellar et al. applied CRISPR/Cas9 to produce PRLR-edited cattle in heat-sensitive Angus and Jersey breeds, providing a valuable model for dissecting the role of this gene in the heat stress response [130]. This study found that PRLR gene edits did not produce observable detriments to male reproductive function, including scrotal circumference, sperm concentration, sperm kinematics, or in vitro fertilizing ability. These null reproductive findings indicate that, while PRLR editing successfully enhanced thermotolerance, its direct benefits to semen quality under heat stress have not yet been demonstrated. Future studies should directly evaluate whether the enhanced thermotolerance conferred by gene editing translates into preserved semen quality during heat exposure. Collectively, these studies underscore the promising application of gene-editing technology in thermotolerant cattle breeding, offering important technical support for the functional validation and genetic improvement of heat stress-related loci. Future research can build upon the established genome-edited thermotolerant bull models by further integrating semen phenotypes, systematically evaluating the protective effects of gene editing against heat stress-induced reproductive damage, thereby linking thermotolerance improvement directly to enhanced semen quality. Nevertheless, the widespread application and adoption of genetically edited livestock have been constrained by safety, technical, and ethical considerations, and thus remain limited at present.

Taken together, nutritional intervention is currently one of the principal strategies for alleviating heat stress in cattle. Although gene-editing-based breeding approaches still confront considerable barriers to practical application, genetic improvement through the selective breeding of thermotolerant sires offers a viable route to achieving long-term, sustainable enhancement of heat tolerance. The organic integration of these two strategies is expected to accelerate the development of thermotolerant breeding bulls, thereby safeguarding the health and sustainable production of cattle herds under heat stress conditions.

## 8. Research Gaps and Future Perspectives

Existing studies have begun to dissect the molecular mechanisms through which heat stress compromises bull semen quality, covering endocrine regulation, oxidative stress, gene expression, and epigenetic modification. Yet most of this evidence derives from single-omics analyses, and the networked, hierarchical architecture underlying heat stress-induced spermatogenic dysfunction has not been systematically delineated. Investigations have typically captured phenotype-associated changes at one molecular level, leaving the temporal order of pathway activation and the synergy among key signaling cascades poorly resolved. The resultant picture of the global regulatory network remains fragmentary.

At the molecular breeding level, numerous candidate genes implicated in spermatogenesis have been identified, but the findings are largely confined to association studies and in vitro validation. The scarcity of systematic in vivo validation in animal models limits the reliability with which these discoveries can be translated into breeding practice. On the intervention front, the beneficial metabolites and probiotics characterized to date still await large-scale experimental confirmation and production-scale application, and the stability, safety, and real-world efficacy of candidate strategies have not been sufficiently assessed. Together, these limitations represent a critical bottleneck that impedes progress from fundamental mechanistic research toward genetic improvement and precision intervention.

To address these bottlenecks, future research should first advance the application of multi-omics integrated analyses in the study of heat stress effects on bull semen quality, by concurrently conducting transcriptomic, metabolomic, epigenomic, and microbiome sequencing to construct cross-layer molecular regulatory networks. In parallel, using modern biotechnological tools, bulls with enhanced thermotolerance could be generated through targeted knock-in or modification of spermatogenesis-related genes. Furthermore, integrating genomic selection with conventional breeding strategies would enable the introgression of identified thermotolerance-associated superior alleles into sire populations, thereby promoting the practical implementation of marker-assisted breeding. With the proposal of the “gut–testis” axis, targeted probiotic intervention has opened a novel non-genetic regulatory avenue for alleviating heat stress-induced reproductive damage. Leveraging the key microbial taxa and metabolites identified through multi-omics, future studies may screen specific probiotic strains with antioxidant, anti-inflammatory, and gut-barrier-maintaining functions, and administer them via feed supplementation or oral formulations to modulate host–microbe interactions. This approach could restore redox balance and the hormonal microenvironment under heat stress, thereby safeguarding bull semen quality.

In summary, multi-omics integration, gene-editing-based improvement, and targeted probiotic intervention are mutually reinforcing and complementary. Their convergence will drive research on heat stress-induced reproductive damage from mechanistic exploration toward the practical translation of genetic improvement and precision management, thereby providing a scientific foundation for enhancing the thermotolerance of breeding bulls in the context of global climate warming.

## 9. Conclusions

Heat stress compromises the semen quality of breeding bulls through a coordinated cascade of biological responses spanning multiple hierarchical levels, from behavioral changes and declines in conventional semen parameters to alterations in the sperm microenvironment, oxidative stress, neuroendocrine disruption, and epigenetic reprogramming. The “gut–testis” axis offers a novel conceptual framework for understanding the full spectrum of heat stress-induced reproductive damage; however, direct mechanistic evidence in bulls remains limited, and current understanding is largely extrapolated from other species. Most studies to date remain anchored in single-omics approaches, leaving the temporal activation and synergistic effects among multiple pathways largely unexplored. Future work should integrate multi-omics datasets to reconstruct the molecular regulatory network governing spermatogenesis under heat stress and establish a multidimensional assessment framework combining genetic, epigenetic, and microbiome signatures. Specifically, it remains to be determined whether these candidate markers can predict: (i) the degree of individual susceptibility to heat stress; (ii) the subsequent deterioration of semen quality; or (iii) actual fertility outcomes. These molecular and microbial indicators remain at the candidate-marker stage; their utility as reliable predictive tools requires prospective temporal validation in independent populations and demonstration of added value beyond conventional indicators such as THI, body temperature, respiratory rate, or routine semen-quality measurements. On this basis, precision nutritional intervention and genetic improvement programs may translate these mechanistic insights into practical strategies for heat tolerance management in breeding bulls under global warming.

## Figures and Tables

**Table 1:** Effects of Probiotics on Semen Quality in Male Animals.

Study (Ref.)	Model	n per Group	Strain(s)	Dose	Route	Duration	Reproductive Indicators
Guo et al. [107]	Mouse (chronic stress model)	6	*Lactobacillus rhamnosus*	3 × 10^10^ cells/mL	Oral	40 days	Sperm count, motility, DNA integrity, ultrastructure
Valcarce et al. [108]	Human (azoospermic patients)	9	*L. rhamnosus CECT8361* + *B. longum CECT7347*	1 × 10^9^ CFU/day	Oral	3–6 weeks	Ejaculate volume, sperm motility, DNA integrity
Mazanko et al. [109]	Cobb male broiler breeders	90	*Bacillus amyloliquefaciens TOA5001*	2.5 × 10^7^ CFU/day	Dietary	6 weeks	Sperm density, proportion of live sperm
Cai et al. [110]	Mouse model of systemic heat stress	10	*Enterococcus faecium* + *Clostridium butyricum*	1 × 10^7^ CFU/day	Oral	7 days	Spermatogenesis, mature spermatozoa in cauda epididymis
Poutahidis et al. [111]	Aging mouse	5–10	*Lactobacillus reuteri ATCC PTA 6475*	3.5 × 10^5^ organisms/mouse/day	Drinking water	Up to 40 weeks	Testicular weight, serum testosterone, Leydig cell proliferation, sperm concentration
Xie et al. [112]	Mouse (CPFX testicular injury model)	10	*Lactobacillus reuteri*	1 × 10^9^ CFU/day	Oral	42 days	Serum testosterone, testis weight

## Data Availability

No new data were created or analyzed in this study. Data sharing is not applicable to this article.
